# Correction: Socioeconomic gradient of lean diabetes in India: Evidence from National Family Health Survey, 2019–21

**DOI:** 10.1371/journal.pgph.0005812

**Published:** 2026-01-08

**Authors:** Surama Manjari Behera, Priyamadhaba Behera, Sanjay K. Mohanty, Rajeev Ranjan Singh, Binod Kumar Patro, Avinaba Mukherjee, Venkatarao Epari

Figs 2 and 3 were uploaded incorrectly. Please see the correct Figs 2 and 3 here.

**Fig 2:**
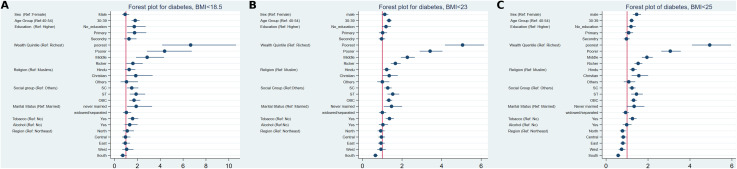
Forest plot of lean type 2 diabetes by BMI cutoff. **A)** <18.5, **B)** <23, and **C)** <25 with socioeconomic variables among females (30–49 years) and males (30–54 years), India, 2019–21.

**Fig 3:**
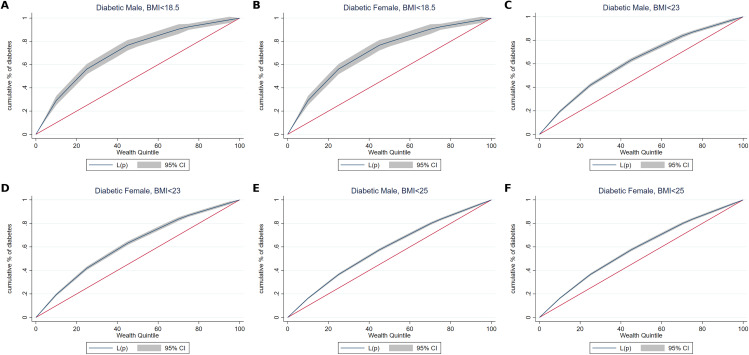
Concentration curve of lean type 2 diabetic among females (30–49 years) and males (30–54 years), India, 2019–21.

The *P* values reported in Tables 5 and 6 were incorrect. Please see the correct Tables 5 and 6 here.

**Table 5:** Concentration index of lean type 2 diabetic among males (30-54 years) and females (30-49 years), India, 2019-21.

BMI	Female	P Value	Male	P Value
**Concentration Index (95% CI)**		**Concentration Index (95% CI)**	
<18.5	-0.42(-0.45, -0.39)	0.001	-0.39(-0.47, -0.31)	0.001
<23	-0.25(-0.26, -0.24)	0.001	-0.21(-0.23, -0.19)	0.001
<25	-0.18(-0.18, -0.17)	0.001	-0.12(-0.14, -0.11)	0.001

**Table 6:** Concentration index of impaired blood glucose level (140-199 mg/dl) among males (30-54 years) and females (30-49 years), India, 2019-21.

BMI	Female	P Value	Male	P Value
**Concentration Index (95% CI)**		**Concentration Index (95% CI)**	
<18.5	-0.45(-0.48, -0.42)	0.00	-0.43(-0.49, -0.35)	0.00
<23	-0.28(-0.30, -0.26)	0.00	-0.24(-0.27, -0.21)	0.00
<25	-0.20(-0.23, -0.18)	0.00	-0.14(-0.17, -0.12)	0.00
