# Supplementary material for: Socioeconomic gradient of lean diabetes in India: Evidence from National Family Health Survey, 2019–21
Source: PLOS Glob Public Health. 2024 May 30;4(5):e0003172. doi: 10.1371/journal.pgph.0003172 (PMC11139297; doi:10.1371/journal.pgph.0003172)
Supplement: S1 Table — (DOCX) [file pgph.0003172.s001.docx]

**S1 Table: Prevalence of lean diabetes among females (15-49 years) and males (15-54) by socio-demographic characteristics, India, 2019-21**

| **Variables** | **Female** | | **Male** | |
| --- | --- | --- | --- | --- |
|  | **Lean Diabetes** | **N** | **Lean Diabetes** | **N** |
| **Age (in Years)** |  |  |  |  |
| <20 | 0.67 | 1,29,931 | 0.11 | 128917 |
| 20-24 | 0.91 | 1,28,755 | 0.13 | 114998 |
| 25-29 | 1.13 | 1,25,477 | 0.13 | 112209 |
| 30-34 | 1.25 | 1,05,603 | 0.20 | 100837 |
| 35-39 | 1.69 | 1,02,254 | 0.33 | 96374 |
| 40-44 | 2.26 | 84,824 | 0.40 | 81945 |
| 45-49 | 2.97 | 88,253 | 0.56 | 83412 |
| 50-54 |  |  | 0.76 | 67403 |
| **Education** |  |  |  |  |
| No education | 1.73 | 1,64,071 | 0.31 | 85012 |
| Primary | 1.68 | 95,206 | 0.35 | 93164 |
| Secondary | 1.33 | 3,85,352 | 0.28 | 453307 |
| Higher | 1.21 | 1,20,466 | 0.26 | 154612 |
| **Wealth Index** |  |  |  |  |
| Poorest | 1.32 | 1,42,053 | 0.24 | 135647 |
| Poorer | 1.54 | 1,52,827 | 0.27 | 152273 |
| Middle | 1.52 | 1,56,552 | 0.29 | 162961 |
| Richer | 1.54 | 1,58,548 | 0.35 | 167241 |
| Richest | 1.28 | 1,55,116 | 0.27 | 167974 |
| **Religion** |  |  |  |  |
| Hindu | 1.43 | 6,19,323 | 0.29 | 639905 |
| Muslim | 1.48 | 1,05,553 | 0.27 | 103510 |
| Christian | 2.07 | 18,329 | 0.39 | 18551 |
| Others | 1.10 | 21,891 | 0.23 | 24129 |
| **Social group** |  |  |  |  |
| SC | 1.49 | 1,70,236 | 0.27 | 172591 |
| ST | 1.12 | 72,929 | 0.24 | 74198 |
| OBC | 1.52 | 3,20,411 | 0.31 | 325645 |
| Others | 1.39 | 2,01,521 | 0.27 | 213661 |
| **Marital status** |  |  |  |  |
| Never married | 0.77 | 1,82,138 | 0.14 | 286051 |
| Married | 1.62 | 5,52,000 | 0.37 | 488138 |
| Widowed/separated | 2.14 | 30,958 | 0.31 | 11905 |
| **Tobacco** |  |  |  |  |
| No | 1.42 | 7,21,131 | 0.25 | 516161 |
| Yes | 1.85 | 43,966 | 0.35 | 269934 |
| **Alcohol** |  |  |  |  |
| No | 1.44 | 7,57,852 | 0.27 | 642355 |
| Yes | 1.23 | 7,244 | 0.37 | 143740 |
| **Region** |  |  |  |  |
| North | 1.06 | 1,08,025 | 0.19 | 116910 |
| Central | 1.27 | 1,91,588 | 0.20 | 197586 |
| East | 1.62 | 1,74,208 | 0.39 | 161459 |
| Northeast | 1.81 | 28,010 | 0.37 | 29030 |
| West | 1.02 | 1,07,704 | 0.18 | 124130 |
| South | 1.95 | 1,55,562 | 0.43 | 156980 |
| **India** | 1.44 | 7,65,096 | 0.29 | 7,86,095 |
