# Supplementary material for: Socioeconomic gradient of lean diabetes in India: Evidence from National Family Health Survey, 2019–21
Source: PLOS Glob Public Health. 2024 May 30;4(5):e0003172. doi: 10.1371/journal.pgph.0003172 (PMC11139297; doi:10.1371/journal.pgph.0003172)
Supplement: S2 Table — (DOCX) [file pgph.0003172.s002.docx]

**S2 Table: State pattern of lean type 2 diabetes, India, 2019-21**

| **State** | **Lean Diabetes (%) (BMI<25)** | **N** |
| --- | --- | --- |
| Jharkhand | 67 | 554 |
| Meghalaya | 66 | 178 |
| Ladakh | 66 | 96 |
| Assam | 64 | 828 |
| Chhattisgarh | 60 | 462 |
| Rajasthan | 59 | 553 |
| Nagaland | 58 | 142 |
| Arunachal Pradesh | 57 | 508 |
| Madhya Pradesh | 56 | 653 |
| West Bengal | 55 | 654 |
| Odisha | 54 | 786 |
| Bihar | 54 | 897 |
| Tripura | 54 | 255 |
| Mizoram | 52 | 176 |
| Uttar Pradesh | 52 | 1,938 |
| Jammu & Kashmir | 51 | 472 |
| Maharashtra | 46 | 625 |
| Sikkim | 45 | 94 |
| Telangana | 44 | 942 |
| Lakshadweep | 43 | 42 |
| Gujarat | 42 | 672 |
| Karnataka | 40 | 879 |
| Kerala | 39 | 652 |
| Tamil Nadu | 39 | 1,851 |
| Haryana | 39 | 596 |
| Himachal Pradesh | 38 | 243 |
| Uttarakhand | 37 | 265 |
| Manipur | 37 | 209 |
| Andhra Pradesh | 35 | 499 |
| Chandigarh | 35 | 40 |
| Andaman & Nicobar Island | 34 | 91 |
| Goa | 33 | 99 |
| Puducherry | 30 | 234 |
| Dadra & Nagar Haveli | 29 | 66 |
| Punjab | 28 | 741 |
| Delhi | 25 | 352 |
| India | 47 | 18,344 |

*Note: State is sorted in descending order of lean diabetes*
