# Supplementary material for: Socioeconomic gradient of lean diabetes in India: Evidence from National Family Health Survey, 2019–21
Source: PLOS Glob Public Health. 2024 May 30;4(5):e0003172. doi: 10.1371/journal.pgph.0003172 (PMC11139297; doi:10.1371/journal.pgph.0003172)
Supplement: Attachment — Submitted filename: Response to Reviwers.docx [file pgph.0003172.s003.docx]

Thank you for considering our work. We have addressed (at our best level) all the issues/comments raised by the journal and reviewers. Still, if there are any issues remain, we will be happy to hear from the journal to further polish the manuscript.

**Journal Requirements:**

1. Please review your reference list to ensure that it is complete and correct. If you have cited papers that have been retracted, please include the rationale for doing so in the manuscript text, or remove these references and replace them with relevant current references. Any changes to the reference list should be mentioned in the rebuttal letter that accompanies your revised manuscript. If you need to cite a retracted article, indicate the article’s retracted status in the References list and also include a citation and full reference for the retraction notice.

Reply: Thank you for the suggestion; we have updated the reference list per the journal format.

2. We noticed you have some minor occurrence of overlapping text with the following previous publication(s), which needs to be addressed:

- <https://doi.org/10.1186/s12909-022-03162-8>
- <https://doi.org/10.1016/j.acalib.2019.02.006>

In your revision ensure you cite all your sources (including your own works), and quote or rephrase any duplicated text outside the methods section. Further consideration is dependent on these concerns being addressed.

Reply: Thank you for the suggestion; we have reviewed the whole manuscript and amended the suggestions.

3. Please send a completed 'Competing Interests' statement, including any COIs declared by your co-authors. If you have no competing interests to declare, please state "The authors have declared that no competing interests exist". Otherwise please declare all competing interests beginning with the statement, "I have read the journal's policy and the authors of this manuscript have the following competing interests:"

Reply: Thank you so much for pointing this out; we have added the competing interest statement in the manuscript as ‘The authors have declared that no competing interests exist’.

4. If you did not receive any funding for this study, please simply state: “The authors received no specific funding for this work.”

Reply: Thank you so much for the suggestion, ‘The authors have not received any funding for this work’. A request letter for waiver was submitted to the financial team of PLOS Global Public Health during the first submission.

5. Please provide separate figure files in .tif or .eps format only and remove any figures embedded in your manuscript file. Please also ensure all files are under our size limit of 10MB.

For more information about figure files please see our guidelines:

<https://journals.plos.org/globalpublichealth/s/figures> 
<https://journals.plos.org/globalpublichealth/s/figures#loc-file-requirement>

Reply: Thank you so much; we have changed all the figures in .tif format

6. Some material included in your submission may be copyrighted. According to PLOS’s copyright policy, authors who use figures or other material (e.g., graphics, clipart, maps) from another author or copyright holder must demonstrate or obtain permission to publish this material under the Creative Commons Attribution 4.0 International (CC BY 4.0) License used by PLOS journals. Please closely review the details of PLOS’s copyright requirements here: PLOS Licenses and Copyright. If you need to request permissions from a copyright holder, you may use PLOS's Copyright Content Permission form.

Reply: Thank you for the suggestion; we have provided the map sources used in the manuscript. <https://onlinemaps.surveyofindia.gov.in/FreeOtherMaps.aspx>

7. Please respond directly to this email or email the journal office and provide any known details concerning your material's license terms and permissions required for reuse, even if you have not yet obtained copyright permissions or are unsure of your material's copyright compatibility.

Potential Copyright Issues:

Fig 1: please (a) provide a direct link to the base layer of the map (i.e., the country or region border shape) and ensure this is also included in the figure legend; and (b) provide a link to the terms of use / license information for the base layer image or shapefile. We cannot publish proprietary or copyrighted maps (e.g. Google Maps, Mapquest) and the terms of use for your map base layer must be compatible with our CC-BY 4.0 license.

Note: if you created the map in a software program like R or ArcGIS, please locate and indicate the source of the basemap shapefile onto which data has been plotted.

If your map was obtained from a copyrighted source, please amend the figure so that the base map used is from an openly available source. Alternatively, please provide explicit written permission from the copyright holder granting you the right to publish the material under our CC-BY 4.0 license.

Please note that the following CC BY licenses are compatible with PLOS license: CC BY 4.0, CC BY 2.0 and CC BY 3.0, meanwhile such licenses as CC BY-ND 3.0 and others are not compatible due to additional restrictions.

If you are unsure whether you can use a map or not, please do reach out and we will be able to help you. The following websites are good examples of where you can source open access or public domain maps: 
* U.S. Geological Survey (USGS) - All maps are in the public domain. ([http://www.usgs.gov](http://www.usgs.gov/)) 
* PlaniGlobe - All maps are published under a Creative Commons license so please cite “PlaniGlobe, [http://www.planiglobe.com](http://www.planiglobe.com/), CC BY 2.0” in the image credit after the caption. (<http://www.planiglobe.com/?lang=enl>) 
* Natural Earth - All maps are public domain. (<http://www.naturalearthdata.com/about/terms-of-use/>)

Reply: Thank you so much for the suggestion, the map was created in ArcGIS using the state shape file from the <https://onlinemaps.surveyofindia.gov.in/FreeOtherMaps.aspx>

**5. Review Comments to the Author**

Please use the space provided to explain your answers to the questions above. You may also include additional comments for the author, including concerns about dual publication, research ethics, or publication ethics. (Please upload your review as an attachment if it exceeds 20,000 characters)

**Reviewer #1**: Dear Authors,

It is an important scientific inquiry that focuses out of the conventional inquiry of diabetes, that is lean DM, which is important for the communities with low BMI (less than overweight), and so, is an important issue in public health. The most literature (more than 50%) have been found within recent within 5 years, written in comprehensible way, and with rational justified. With suffice sample, the data analysis has been carried out in accordance with scientific rigour. Descriptive analysis along with logistic regression, the concentration index with their 95% CI, have been reported. The figures added further understanding of the DM distribution and inequality variations among different population segments. However, the following are commented on:

Reply: Thank you very much for your appreciation and suggestion

1. Despite having analysis and results reported clearly, the discussion looks weaker in comparison. Although lean DM and its associated factors have been discussed clearly, its pre/sub-clinical situation, which might be important from pathogenetic dimensions and thus screened earlier, impacting higher cost-effectiveness, and even more important from the UHC. Taken as example, the **pre-HTN and pre-clinical scenario have been missed, as these have been found with opposite relationship with the obvious HTN and such other** chronic diseases. When the HTN was found to be higher among the richer segments, the Pre-HTN was significantly higher with C~-0.18 among the poor and, how these would impact the UHC, has been clearly explored (cited in our review article: Adhikari et al. Need for HTA supported risk factor screening for hypertension and diabetes in Nepal: A systematic scoping review). This is further supported as in your results that the inequalities (C values) towards higher BMI are further decreased, as compared to the lower BMIs. This may indicate that the pre-DM situation might be higher among with the lower BMIs, even lower than 18.5, which may coincide and be a fate of the poor. So, I recommend adding further discussion regarding the pre-DM scenario with the values of C in India, and even among the countries with similar socio-economic statuses.

Reply: Thank you so much. We have rewritten the whole discussion section based on the suggestions. We have also generated a table on impaired blood glucose levels (140-199mg/dl) and added it to the manuscript as 5b. Also, we have provided the population-based prevalence of lean diabetes as supplementary material.

2. Logistic regression is seemed to have been calculated only for raw odds ratios. These could be further calculated for adjustment, which may add further importance of associated factors, with further determination.

Reply: Thank you so much for the suggestion. The regression was adjusted for background characteristics such as age, sex, caste, level of education, wealth quintile, region, etc. A note of the same is added below the table in the manuscript.

3. Concentration indexes have been reported with 95% CI though, it would be better to further add the p-values, which may add easiness in interpretations for the readers.

Reply: Thank you so much for the suggestion; we have added the P values for the concentration index in the manuscript.

4. Finally, Some words are found joined together, however, this may have been gotten during the pdf conversion process, still hopefully, attentions would be given.

Reply: Thank you so much for the suggestion. We have gone through the whole manuscript and adjusted for these errors.

5. It is further hypothesized with undiagnosed DM, which is missed, in addition to the Pre-DM, that is mentioned. So, hope to be discussed in that direction, also.

Reply: Thank you so much for the suggestion. We have estimated the pre diabetic scenario and discussed it in the result and discussion section of the revised manuscript

**Reviewer #2:** Very good article that highlights the findings on an important health issue.

Providing my suggestions to improve the quality of the paper as follows:
1. writing need more correction (no spaces between many words), more clear especially in "results" section

Reply: Thank you so much for the suggestion we gone through the whole manuscript and adjusted for these errors.

2. page 14, 1st paragraph: (Among all males with diabetes (N=3140)...), is a little bit confusing, using the word "under"

Reply: Thank you so much for the suggestion, we have reframed the lines in revised manuscript.

3. BMI group (18.5 - 23) figure is not right in the first line) please rephrase to make it more clear

Reply: Thank you so much for the suggestion, we have reframed the lines in revised manuscript

4. Table 3 & forward: for lean diabetes stratification by BMI index, are the categories cumulative or discrete ones like this (<18.5, then 18.5 - <23, and 23 - < 25)? please, clarify and make it more clear

Reply: Thank you so much for the suggestion we have added a note for the categorisation of BMI in the manuscript. Categorisation of BMI cutoffs is continuous in nature.

5. in table 4, define the "asterisks**" meaning, the p=values , to be more understandable and clear.

Reply: Thank you for the pointing out, we have added a note for the same below the table.

6. Please, properly cite the "data set" used in this survey, as instructed in the original website as in this link " <https://dhsprogram.com/publications/Recommended-Citations.cfm> "

Reply: Thank you so much for the suggestion, we have gone through the several publications to check the citation, even from the Plos global public health journal, and we find the similar ways of citation as we have already done. For cross check one may refer to these publications from NFHS 5 in Plos global public health:

<https://journals.plos.org/globalpublichealth/article?id=10.1371/journal.pgph.0002117>

<https://journals.plos.org/globalpublichealth/article?id=10.1371/journal.pgph.0000205>

<https://journals.plos.org/globalpublichealth/article?id=10.1371/journal.pgph.0000243>
